# Potential influences of expression levels of MFGE8 and HMGB1 on the intestinal mucosal barrier function and inflammatory response after blunt abdominal injury in rats

**DOI:** 10.1590/acb370303

**Published:** 2022-06-01

**Authors:** Lijun Tao, Hongbo Xu, Qianggui He

**Affiliations:** 1 MD. The First Affiliated Hospital of Wenzhou Medical University – Department of Trauma Surgery (Emergency Surgery) – Wenzhou, China.

**Keywords:** Abdominal Injuries, rho-Associated Kinases, HMG-Box Domains, Rats

## Abstract

**Purpose:**

To explore the influence of milk fat globule-EGF factor 8 protein (MFGE8) on blunt abdominal injury in Sprague Dawley (SD) rats through the RhoA/ROCK signaling pathway.

**Methods:**

The blunt abdominal injury model was generated in SD rats. A total of 44 rats was randomly assigned into three groups. Rat blunt abdominal injury was assessed by the abbreviated injury scale (AIS). The rats were sacrificed for observing the morphology of the abdominal cavity and intestines. Hematoxylin and eosin staining was performed to visualize the pathological changes of rat intestines. Positive expressions of MFGE8 and high mobility group box 1 (HMGB1) in rat intestines were examined by immunohistochemical staining. Protein levels were determined by Western blot. Serum levels of tumor necrosis factor α (TNF-α), IL-1β, IL-6 and malondialdehyde (MDA) were measured by enzyme linked immunosorbent assay (ELISA).

**Results:**

Blunt abdominal injury resulted in inflammatory response of intestinal tissues, increased serum levels of TNF-α, IL-1β, IL-6 and MDA, upregulation of HMGB1, RhoA and ROCK2, and downregulation of MFGE8 in rats, which were significantly alleviated by intervention of rhMFGE8.

**Conclusions:**

MFGE8 protects the intestinal mucosal barrier function after blunt abdominal injury in rats by downregulating HMGB1. Moreover, it alleviates inflammatory response and oxidative stress caused by blunt abdominal injury in rats through downregulating RhoA and ROCK.

## Introduction

Blunt abdominal injury is prevalently seen in the general surgery department, usually caused by production, life, and traffic accidents. It is featured by a sudden onset, rapid progression, and poor prognosis^1^. Following the liver and spleen, the intestines and mesenterium are the third most-common damaged tissues by blunt abdominal injuries^2^. Recent evidences have shown that the intestinal mucosal barrier dysfunction exerts a bridge function in the development of systemic inflammatory response syndrome (SIRS) and multiple organ dysfunction syndrome (MODS)^3^. Therefore, clarifying the pathogenesis of intestine damages following blunt abdominal injury contributes to alleviate intestinal mucosal barrier dysfunction and improve the prognosis.

Milk fat globule-EGF factor 8 protein (MFGE8) is a lipophilic glycoprotein located on the cell membrane. Abundant studies have proven that MFGE8 is a bridge linking apoptotic cells and phagocytic cells, which is responsible for clearing apoptotic cells in affected tissues^4^. In addition to the function of clearing apoptotic cells, MFGE8 also participates in various physiological and pathological events. It reduces the infiltration of neutrophils in the lung by regulating CXCR212^5^ and inhibits tissue fibrosis by stimulating the removal of collagens from inflammation tissues^6^. MFGE8 is capable of repairing intestinal mucosa through alleviating intestinal mucosal inflammation and promoting the migration of intestinal mucosal epithelial cells^7^.

Wang et al.^8^ demonstrated that the balance between expression levels of MFGE8 and high mobility group box 1 (HMGB1) determines the endocytosis of macrophages and other pathological influences induced by chronic alcohol consumption. Serving as an important inflammation mediator, HMGB1 activates TGF-β1 through the p38 MAPK and RhoA signaling pathways via inducing the release of IL-1β^9^. However, potential influences of MFGE8 and HMGB1 on blunt abdominal injury remain largely unclear. In the present study, we generated a blunt abdominal model in rats, and explored the function of MFGE8 in influencing pathological changes of intestines and HMGB1 expression through intravenous injection of rhMFGE8 in rats.

## Methods

### Reagents

The recombinant human MFGE8 (LC11NO2901) was from Sino Biological Inc.; hematoxylin stain (19120302) and peroxidase-conjugated goat anti-rabbit IgG (H+L) antibody (ZB-2301) were from ZSGB-BIO; eosin stain (E607321) was from Sangon Biotech; ultra-clear advanced mounting resin was from BaSO Biotech; Scott bluing solution (G1865) and bovine serum albumin (BSA, A8020) were from Solarbio; HMGB1 polyclonal antibody (10829-1-AP) was from Proteintech; anti-MFGE8 polyclonal antibody (PA5-109955) was from Invitrogen; the diaminobenzidine (DAB) staining kit (CW0125), neutral resin (CW0136) and BCA protein assay kit (CW0014S) were from CWBIO; rat IL-1β kit (MM-0047R1), rat IL-6 kit (MM-0190R1), rat tumor necrosis factor α (TNF-α) kit (MM-0180R1), and rat malondialdehyde (MDA) kit (MM-0385R1) were from Meimian, China; enhanced chemiluminescence (ECL) Western blot substrate (RJ239676) and Western blot marker (#26617) were from ThermoFisher; polyvinylidene difluoride (PVDF) membranes (IPVH00010) were from Millipore; and skimmed milk powder (P1622) was from Applygen.

The microscope (BX43) was from Olympus; rotary microtome (2235) was from Leica; electric blast drying oven (HGZF-101-1)was from Shanghai Yuejin; pressure cooker (YS20ED) was from Supor; induction cooker (HK-22) was from Hanke, Zhongshan; the automatic microplate spectrophotometer (WD-2102B) and the vertical electrophoresis apparatus (DYY-6C) were from Beijing LIUYI Biotech; and the constant temperature incubator (DHP-9054) was from Biobase.

### Blunt abdominal injury model in rats

**Figura 1:**
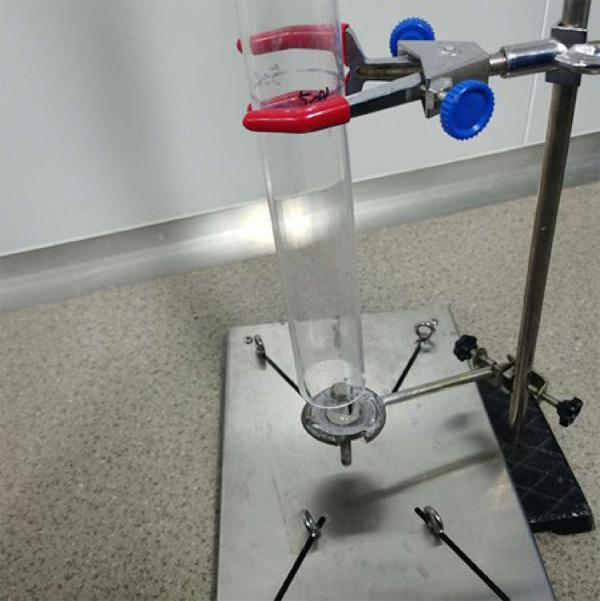
Modified BIM-IV bio-impact machine (250-g metal ball).

Female 8-week-old Sprague Dawley (SD) rats in Specific Pathogen Free (SPF) level weighing 180-220 g were provided by Hunan SJA Laboratory Animal Co., Ltd. (Certificate No., SCXK, Hunan, 2019-0004). A total of 44 female 8-week-old SD rats in SPF level weighing 180-220 g were randomly assigned into control group (n = 5), model group (n = 22) and model+ rhMFGE8 group (n = 17). They had free access to water, but food was forbidden for 12-h fasting. After weighing, rats were anesthetized by intraperitoneal injection of 10% chloral hydrate (2.8 mL/kg). Rats in control group were fixed for 45 min, and then placed into cages. The hair on the abdomen and back of rats in model group and model + rhMFGE8 group was shaved off. A self-made modified BIM-IV bio-impact machine was prepared for simulating blunt abdominal injury (Fig. 1).

**Figura 2:**
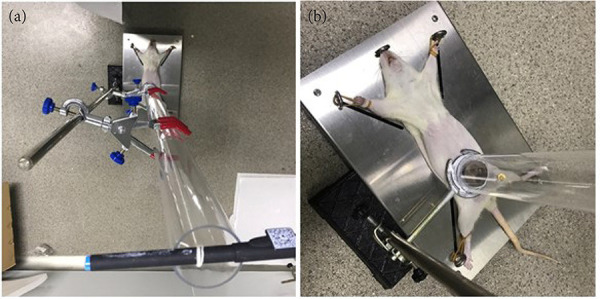
Establishment of trauma model.

Briefly, a self-made vertical line was used to determine the vertical position of the guide tube. After fixation of rats, a 250-g metal ball freely fell from the top of the bio-impact machine to impact on the left of lower abdomen with an impact area of 1 cm^2^ (Fig. 2). Parenchymal organs and the spine should be avoided. Intravenous injection of rhMFGE8 was performed in rats of model + rhMFGE8 group prior to the impact injury.

Blunt abdominal injury of rats was assessed according to the abbreviated injury scale (AIS) scores. After sacrifice at 4 and 24 h, intestinal rupture, contusion, hematoma of mesenterium, hemoperitoneum, and damages of abdominal parenchymal organs were recorded in detail. Damaged intestine tissues and serum samples were collected for the following experiments. Hematoxylin and eosin (H&E) staining was performed to visualize the pathological changes of rat intestines. Positive expressions of MFGE8 and HMGB1 in rat intestines were examined by immunohistochemical staining. Protein levels of HMGB1, MFGE8, RHoA and ROCK2 in rat intestinal mucosa were determined by Western blot. In addition, serum levels of TNF-α, IL-1β, IL-6 and MDA in rats were measured by enzyme linked immunosorbent assay (ELISA).

All animal procedures were approved by Laboratory Animal Ethics Committee of Wenzhou Medical University & Laboratory Animal Centre of Wenzhou Medical University (Approval ID: wydw2020-0844).

### H&E staining

Intestine tissues were washed in running tap water, and dehydrated in 70, 80, and 90% ethanol. After deparaffinization in the mixture solution containing 100% ethanol and xylene with an equal volume for 15 min, tissues were later incubated in xylene I for 15 min, and xylene II for another 15 min. Subsequently, they were induced in the mixture solution containing xylene and paraffin with the equal volume for 15 min, and permeabilized in paraffin I and II for 50-60 min each. After paraffin-embedded and slice, tissue sections were dewaxed, washed, and stained in hematoxylin solution for 3 min. After 15-s differentiation in acid alcohol and 15-s blueing up, sections were washed in tap water, and counterstained with eosin solution for 3 min. Sections were finally washed, dehydrated, cleared, and mounted for observation under a microscope.

### ELISA

Serum samples of rats were collected for detecting IL-1β, IL-6, TNF-α and MDA following recommendations of commercial kits by ELISA.

### Western blot

Intestine tissues were lysed on ice, and the lysate mixture was centrifuged at 12,000 r/min for 10 min. The supernatant was transferred to a new Eppendorf tube, and preserved at -20º. After measurement of protein concentrations using the BCA protein assay kit, samples adjusted to the same concentration were denaturized, separated by SDS-PAGE for 1.5 h and transferred on PVDF membranes at 300 mA. After incubation with primary antibodies at 4º overnight, and secondary antibodies at room temperature for 2 h, membranes were washed, and visualized using the ECL reagents. Grey value of targeted protein was measured using the Image-Pro Plus.

### Immunohistochemical staining

Paraffin-embedded tissue sections were dewaxed, incubated in citrate buffer for antigen retrieval, heated for 2 min in a pressure cooker and naturally cooled down. After phosphate-buffered saline (PBS) washing, sections were placed in a wet box at room temperature, in which freshly prepared 3% H_2_O_2_ was added to quench endogenous peroxidase activity. Ten minutes later, sections were washed in PBS (5 min × 3), and any excess reagent was wiped away. After blockage in 5% BSA at 37°C for 30 min, sections were incubated with primary antibodies [anti-HMGB1 (1:200), anti-MFGE8 (1:150)] at 4°C overnight, and peroxidase-conjugated goat anti-rabbit IgG (H+L) antibody (1:150) at 37°C for 30 min, sections were stained with DAB solution, counterstained with hematoxylin, washed in tap water, mounted and observed under a microscope.

### Statistical analyses

Statistical Package for the Social Sciences (SPSS) 20.0 was used for statistical analyses. Quantitative data were expressed as mean ± standard deviation (x ± s) from three replicates. Their differences between groups were compared by the independent sample Student’s t-test, while those among three or more groups were compared by one-way analysis of variance (ANOVA), followed by the Student-Newman-Keuls (SNK) method for stepwise multiple comparison. P < 0.05 was considered as statistically significant.

## Results

**Figura 3:**
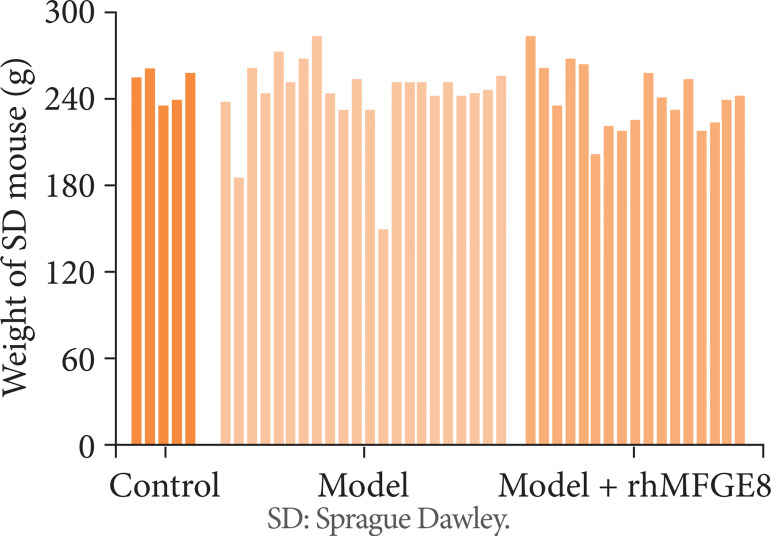
Distribution of rat body weight in control group, model group and model + rhMFGE8 group.

There was no significant difference in rat body weight after modeling among the three groups (Fig. 3, P > 0.05). The numbers of death, and rats with intestinal rupture, contusion, and abdominal parenchymal organ injury (liver) were similar between model group and model + rhMFGE8 group. No cases of intestinal rupture were reported in model + rhMFGE8 group. In addition, the numbers of rats with hematoma of mesenterium and hemoperitoneum were lower in model + rhMFGE8 group than those of model group (Table 1).

**Table 1:** Rat damages after blunt abdominal injury.

Group	Control group (n = 5)	Model group (n = 22)	Model + rhMFGE8 group (n = 17)
Average body weight (g)	251	244	241
Significant difference in body weight	None		
Death (n)	0	3	2
Intestinal rupture (n)	0	1	0
Contusion (n)	0	9	10
Hematoma of mesenterium (n)	0	7	5
Hemoperitoneum (n)	0	17	14
Abdominal parenchymal organ injury (liver, n)	0	2	1

**Figura 4:**
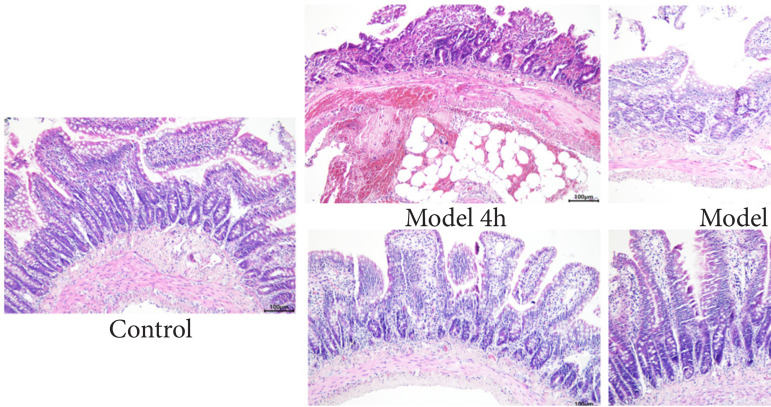
Hematoxylin and eosin staining of rat intestine tissues (magnification = 200×).

H&E staining of rat intestines showed a normal intestinal structure with orderly arranged intestinal villi, with the absence of necrosis in control group. At 4 h of modeling, disorderly arranged intestinal villi, abundant infiltration of inflammation cells, and obvious intestinal wall congestion were observed in model group. More seriously, necrosis and abscission of intestinal villi were seen at 24 h of model group. The abovementioned pathological changes of rat intestines were significantly alleviated by rhMFGE8 intervention, manifesting as milder inflammation, less abscission of intestinal villi, and a relatively normal intestinal structure (Fig. 4). It is suggested that MFGE8 exerted an anti-inflammation function in intestines of rats with blunt abdominal injury.

**Figura 5:**
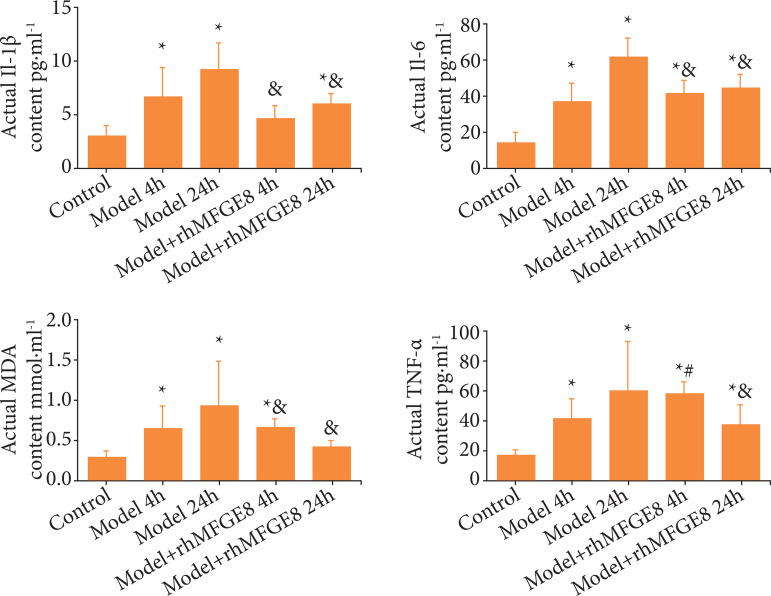
Relative levels of TNF-α, IL-1β, IL-6, and MDA in rat serum detected by ELISA.

MDA: malondialdehyde; TNF-α: tumor necrosis factor α; ELISA: enzyme linked immunosorbent assay; *p < 0.05, control group *vs*. the remaining groups;^#^p < 0.05, model group at 4 h *vs*. model + rhMFGE8 group; ^&^p < 0.05, model group at 24 h *vs*. model + rhMFGE8 group.

Previous evidences have supported the close relationship between intestinal mucosal barrier dysfunction, and oxygen radicals, cytokines and inflammatory factors. In the present study, relative levels of TNF-α, IL-1β, IL-6, and MDA were significantly higher in model group than those of control group, which achieved the peak levels at 24 h of modeling. Significantly lower levels of TNF-α, IL-1β, IL-6 and MDA were detected at 24 h of modeling in model + rhMFGE8 group than those of model group (Fig. 5). It was concluded that blunt abdominal injury in rats significantly enhanced relative levels of TNF-α, IL-1β, IL-6 and MDA, which could be inhibited by rhMFGE8 intervention.

**Figura 6:**
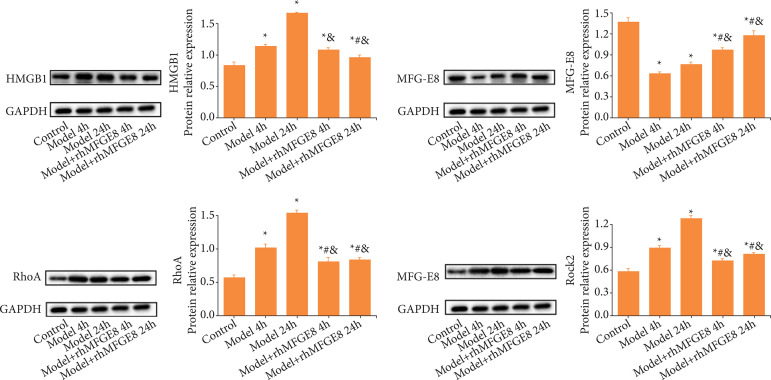
Protein levels of HMGB1, MFGE8, RhoA and ROCK2 in rat intestines detected by Western blot.

HMGB1: high mobility group box 1; GAPDH: Glyceraldehyde-3-Phosphate Dehydrogenase;; MFGE8: milk fat globule-EGF factor 8 protein; *p < 0.05, control group *vs*. the remaining groups; ^#^p < 0.05, model group at 4 h *vs*. model + rhMFGE8 group; ^&^p < 0.05, model group at 24 h *vs*. model + rhMFGE8 group.

It is reported that HMGB1 is a vital inflammatory factor, and the RhoA/ROCK2 signaling pathway is involved in the inflammatory response and oxidative stress. Here, we detected their protein levels in rats by Western blot. Compared with control group, HMGB1, RhoA and ROCK2 were upregulated in model group, and peaked at 24 h of modeling. However, their protein levels were significantly lower in model + rhMFGE8 group than those of model group at either 4 or 24 h. Protein level of MFGE8 was significantly lower in model group than that of control group (Fig. 6). It was concluded that MFGE8 could downregulate HMGB1, RhoA and ROCK2, thus against inflammatory response and oxidative stress in rats caused by blunt abdominal injury.

**Figura 7:**
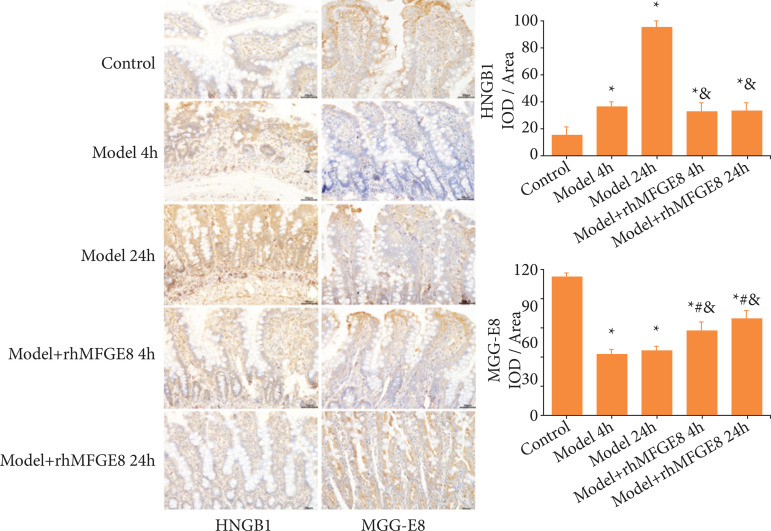
Positive expressions of MFGE8 and HMGB1 in rat intestines detected by immunohistochemical staining.

HMGB1: high mobility group box 1; MFGE8: milk fat globule-EGF factor 8 protein; *p < 0.05, control group *vs*. the remaining groups;^#^p < 0.05, model group at 4 h *vs*. model + rhMFGE8 group; ^&^p < 0.05, model group at 24 h *vs*. model + rhMFGE8 group.

To further validate our findings, positive expressions of HMGB1 and MFGE8 in rat intestines were also examined by immunohistochemical staining. Consistently, positive expression of HMGB1 was significantly higher in model group than that in control group, while the MFGE8 was lower. Their positive expressions were significantly reversed by rhMFGE8 intervention (Fig. 7). Collectively, HMGB1 and MFGE8 were involved in intestinal mucosal barrier dysfunction following blunt abdominal injury, and MFGE8 protected it through regulating the RhoA/ROCK signaling pathway.

## Discussion

The majority of abdominal injuries belong to blunt trauma, rather than penetrating trauma^10^, and blunt intestinal injury and mesenteric injury frequently occur in severe blunt abdominal injury cases^11^^,^ 12. In the present study, the blunt abdominal injury model in rats was generated by free fall of a 250-g metal ball from the top of the bio-impact machine to impact on rat abdomen. After administration of rhMFGE8 in rats, AIS score of blunt abdominal injury and pathological conditions of damaged intestines were significantly improved.

MFGE8 is a vital regulator in the recovery of intestinal mucosal barrier function through stimulating the interaction among multiple types of cells, including intestinal epithelial cells. Cell proliferation, differentiation and migration are essential events for maintaining the integrity of the epithelial layer. Bu et al.^7^ validated that MFGE8 promotes the migration of intestinal epithelial cells through the reorientation of actin cytoskeleton, and the deficiency of MFGE8 blocks the migration of intestinal epithelial cells, impairs the recovery ability, and suppresses mucosal healing in septic mice. MFGE8 is also proven to be beneficial to colitis and other intestinal damages^13^^-^ 15.

Our findings showed that the number of rats with hematoma of mesenterium and hemoperitoneum was lower in model + rhMFGE8 group, and the pathological conditions of rat intestines were significantly improved at 4 and 24 h of rhMFGE8 intervention based on AIS scores and H&E staining images. In addition, MFGE8 also exerts a regulatory effect on inflammatory response.

A growing number of studies have shown the remarkable change of MFGE8 expression in intestinal mucosa of enteritis mice, presenting an obvious anti-inflammation function^16^. Through mediating inflammatory response and accumulated apoptotic cells during the tendon repair process, MFGE8 promotes the healing of injured tendon tissues^17^. Last et al.^18^ revealed that MFGE8 is capable of alleviating renal ischemia reperfusion injury through regulating protein levels of IL-6 and TNF-α. The damage of intestinal mucosal barrier is closely linked to oxygen radicals, cytokines and inflammatory mediators^19^^,^ 20. Consistently, intervention of rhMFGE8 in our study significantly inhibited the upregulation of IL-1β, IL-6 and MDA following blunt abdominal injury in rats.

HMGB1 is a member of the high mobility group, showing a critically important role in regulating innate and adaptive immune response^21^. The close interaction between MFGE8 and HMGB1 has been previously reported. For example, downregulated MFGE8 and upregulated HMGB1 are detected in mice with chronic obstructive pulmonary disease^22^. The HMGB1/MFGE8 axis is of great significance in the pathogenesis of acute pancreatitis^23^. Although we did not analyze the targeting relationship between MFGE8 and HMGB1 in the present study, both Western blot and immunohistochemical staining results supported the negative regulation between expression levels of MFGE8 and HMGB1 in intestine tissues of rats with blunt abdominal injury. The RhoA/ROCK2 signaling pathway can be activated by inflammatory response and oxidative stress^24^^,^ 25. In atherosclerosis, activation of HMGB1 and RhoA/Rac1 is closely related to vascular inflammation induced by NF-κB phosphorylation in endothelial cells^26^.

This study showed downregulation of HMGB1, RhoA and ROCK2 in rats of model + rhMFGE8 group, suggesting that MFGE8 could inhibit expression levels of them. However, the regulatory relationship between HMGB1 and the RhoA/ROCK2 signaling pathway was not investigated in the present study, which requires further explorations.

## Conclusions

In conclusion, overexpression of MFGE8 through intravenous injection of rhMFGE8 in rats with blunt abdominal injury significantly protects the intestinal mucosal barrier function and pathological changes of intestines. In addition, overexpressed MFGE8 is conductive to alleviate inflammatory response and oxidative stress induced by blunt abdominal injury through downregulating HMGB1, RhoA and ROCK. Our findings provided a novel target for the diagnosis and treatment of intestine injuries.
